# The novel complex combination of alum, CpG ODN and HH2 as adjuvant in cancer vaccine effectively suppresses tumor growth *in vivo*

**DOI:** 10.18632/oncotarget.17504

**Published:** 2017-04-28

**Authors:** Yaomei Tian, Meng Li, Chaoheng Yu, Rui Zhang, Xueyan Zhang, Rong Huang, Lian Lu, Fengjiao Yuan, Yingzi Fan, Bailing Zhou, Ke Men, Heng Xu, Li Yang

**Affiliations:** ^1^ State Key Laboratory of Biotherapy, West China Hospital, Sichuan University and Collaborative Innovation Center, Chengdu, Sichuan, China; ^2^ Chengdu Blood Center, Chengdu, Sichuan, China

**Keywords:** combinatorial adjuvant, aluminum salts, CpG oligodeoxynucleotide, innate defense regulator peptide HH2, anti-tumor immune responses

## Abstract

Single-component adjuvant is prone to eliciting a specific type of Th1 or Th2 response. So, the development of combinatorial adjuvants inducing a robust mixed Th1/Th2 response is a promising vaccination strategy against cancer. Here, we describe a novel combination of aluminum salts (alum), CpG oligodeoxynucleotide (CpG) and innate defense regulator peptide HH2 for improving anti-tumor immune responses. The CpG-HH2 complex significantly enhanced the production of IFN-γ, TNF-α and IL-1β, promoted the uptake of antigen and strengthened the activation of p38, Erk1/2 and NF-κB *in vitro*, compared to CpG or HH2 alone. Immunization with NY-ESO-1 antigen plus alum-CpG-HH2 combinatorial adjuvant effectively inhibited tumor growth and reduced tumor burden in prophylactic and therapeutic tumor models and even in passive serum or cellular therapy. In addition, co-administration of NY-ESO-1 with alum-CpG-HH2 combinatorial adjuvant markedly activated NK cell cytotoxicity, induced antibody-dependent cellular cytotoxicity (ADCC), dramatically elicited cytotoxic T lymphocytes (CTLs) response, and increased infiltrating lymphocytes in tumors. Moreover, *in vivo* depletion of CD8^+^ T cells completely and depletion of NK cells partially blocked the anti-tumor activity of NY-ESO-1-alum-CpG-HH2 immunization. Overall, our results demonstrate a novel adjuvant combination for cancer vaccine with efficient immunomodulation by stimulating innate immunity and mediating adaptive immunity.

## INTRODUCTION

Among the enormous approaches for cancer immunotherapy, cancer vaccines are strongly encouraged and highly advanced for its potential benefit in clinic [1]. However, several clinical trials have revealed that tumor-associated antigens in cancer vaccines share weak immunogenicity or poor ability to induce effective cytotoxic T cells (CTLs) and anti-tumor immune responses [2]. Thus, there is still a great need of adjuvants in cancer vaccines for eliciting an efficient and long-lasting anti-tumor immunity [3]. Adjuvants possessing both immunomodulatory effect and enhanced delivery capabilities are essential features for vaccines to generate adequate humoral and cellular immune responses [4]. Addition of adjuvants to certain vaccines enhances the magnitude and durability of antigen-specific immune responses [5]. Yet, a single adjuvant often results in either no response or weak anti-tumor activity. Therefore, development of multiple or combinatorial adjuvants that can induce a broader humoral immune response and desired CTLs is a promising approach for design of effective cancer vaccines [6, 7].

Recently, some vaccines incorporating combinatorial adjuvants have been well studied in pre-clinical or clinical settings. For example, AS04 adjuvant combination(alum+MPL) is employed in two licensed HPV vaccines, Cervarix and Gardasil, to induce higher titers of antibodies [8]. Alum, the unique adjuvant approved by the U. S. Food and Drug Administration (FDA), can sharply augment antigen persistency, antigen uptake and local immunostimulatory response[9]. Recent studies have gradually revealed that alum activates the NALP3 inflammasome followed by the release of IL-1β, IL-18, IL-33, and induces a Th2-biased immune response [10, 11]. However, poor Th1-type immune response induced by alum makes it inappropriate as a single component in cancer vaccines.

Innate defense regulator peptides (IDRs), synthetic mimics of host defense peptides, have weak antimicrobial activity, but have preferable ability to modulate the immune response [12]. HH2 (VQLRIRVAVIRA-NH2) is one of such IDRs, which has recently been shown to induce the production of MCP-1 [13], modulate human neutrophil functions [14] and suppress bacterial infection via enhancing chemokine induction and cell recruitment [15]. Remarkably, HH2 has demonstrated a promising function as a component of vaccine adjuvants. Our previous studies have shown that HH2 improves the adjuvant activity of alum-polysaccharide complex [16]. Alum-polysaccharide-HH2 combinational adjuvant effectively stimulates protective cellular immune responses and anti-tumor immunity [16]. Furthermore, in response to the combination of alum and HH2, the Th1-type immune response is improved, while the Th2-type immunity persists predominantly [16].

CpG-ODN, triggering a Th1-type immune response *via* interaction with Toll-like receptor 9 (TLR9), has gained considerable interests as a candidate immunomodulator for therapeutic application against tumors [17–20]. The adjuvant combination composed of CpG and other adjuvants has been applied to activate CD8^+^ T cells in clinical trials of melanoma vaccines [21, 22]. Recently, the enhanced adjuvant activities of CpG-HH2 complex have been reported. CpG synergizes with HH2 to activate innate immune responses [23, 24] and link innate and adaptive immunity [25]. Our previous work has shown that HH2 changes the type of immune responses induced by alum-CpG combination and reduces the side effect induced by CpG. Moreover, compared with alum alone, alum-CpG-HH2 combinatorial adjuvant results in excellent humoral immunity, enhances T cell proliferative response and especially balances Th1/Th2 immune response [26]. Based on the above findings, we hypothesized that the combination of alum, CpG and HH2 could avoid moderate efficacy or side effect caused by a single adjuvant and induce a mixed Th1/Th2 response.

In this report, we present evidence showing the activity of alum-CpG-HH2 combinatorial adjuvant in cancer vaccine. Immunization with NY-ESO-1 and alum-CpG-HH2 combinational adjuvant significantly enhanced Th2-type immune response and especially induced desired Th1-type immune response. Importantly, the tumorigenesis and growth of tumors in mice were dramatically suppressed by co-administration of NY-ESO-1 with alum-CpG-HH2 combinatorial adjuvant.

## RESULTS

### The CpG-HH2 complex induces secretion of cytokines, promotes the uptake of antigen, and enhances the activation of p38, Erk1/2 and NF-κB *in vitro*

**Figure 1:**
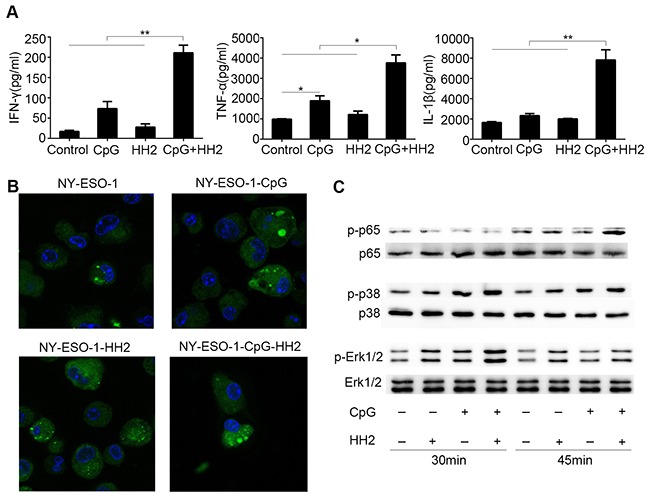
The effects of CpG-HH2 complex *in vitro* (**A**) Human PBMCs (5×10^5^) were stimulated with CpG, HH2 or CpG-HH2 adjuvant complex for 24 h. Then the supernatants were collected and used for cytokines detection by ELISA. Error bars represent mean + SEM. *p < 0.05, **p < 0.01. (**B**) BMDCs were incubated with Alexa Fluor 488-conjugated native NY-ESO-1 (green) alone or protein adjuvant complex for 3 h. Cells were subsequently fixed, stained with DAPI (blue) and detected by confocal laser microscopy. (**C**) Western blot analysis of p-p65, p-p38, and p-Erk1/2 expression.

To assess the immunostimulatory effect of the CpG-HH2 complex, first we analyzed the expression levels of three key immune regulators: IFN-γ, TNF-α and IL-1β, in PBMCs. For this, human PBMCs were stimulated with CpG, HH2 or CpG-HH2 complex for 24 h, followed by ELISA analysis. A synergistic effect was concluded if the expression levels of the cytokines induced by CpG-HH2 complex were at least 2-fold higher compared with the summed cytokines released by CpG alone or HH2 alone. Of note, CpG-HH2 complex resulted in a higher level of IFN-γ (211.1±18.7 pg/ml) and showed a synergistic effect (CI = 2.3±0.4). IL-1β (7,821.5±1,429.1 pg/ml) and TNF-α (3,766.5±389.6 pg/ml) were significantly increased by CpG-HH2 complex, compared with CpG alone or HH2 alone, but did not show a synergistic effect (CI = 1.8 for IL-1β; CI = 1.2 for TNF-α) (Figure 1A). The results suggest that CpG-HH2 complex has better immunostimulatory effect than CpG or HH2.

To investigate whether CpG-HH2 complex adjuvant had an effect on the function of DCs, antigen uptake was examined. To this end, BMDCs were incubated with Alexa Fluor 488-conjugated native NY-ESO-1 alone or protein adjuvant complex (NY-ESO-1-CpG, NY-ESO-1-HH2 or NY-ESO-1-CpG-HH2) for 3 h, followed by DAPI staining and confocal laser microscopy. By semi-quantification with ImageJ software, the mean fluorescence intensities of all groups were: 14,544.5 for NY-ESO-1 alone; 56,348 for NY-ESO-1 plus CpG; 18,394.4 for NY-ESO-1 plus HH2; 210,011.5 for three component complex NY-ESO-1-CpG-HH2 (Figure 1B). As expected, the mean fluorescence intensity value of NY-ESO-1-CpG-HH2 treatment was the highest in all the groups and displayed a synergistic effect (CI=2.8).

To identify the signaling pathways activated by CpG-HH2 complex, the phosphorylation of NF-κB (p65) and p38 and Erk1/2 was examined using Western blotting. As shown in Figure 1C, CpG-HH2 complex was found to considerably induce the phosphorylation of p65 in BMDCs following stimulation for 45 min. Erk1/2 and p38 were also remarkably phosphorylated when BMDCs were incubated with CpG-HH2 complex for 30 min. These results suggest that CpG-HH2 complex has desired properties to be a promising adjuvant.

### Alum-CpG-HH2-NY vaccine effectively suppresses tumor growth in melanoma models

**Figure 2:**
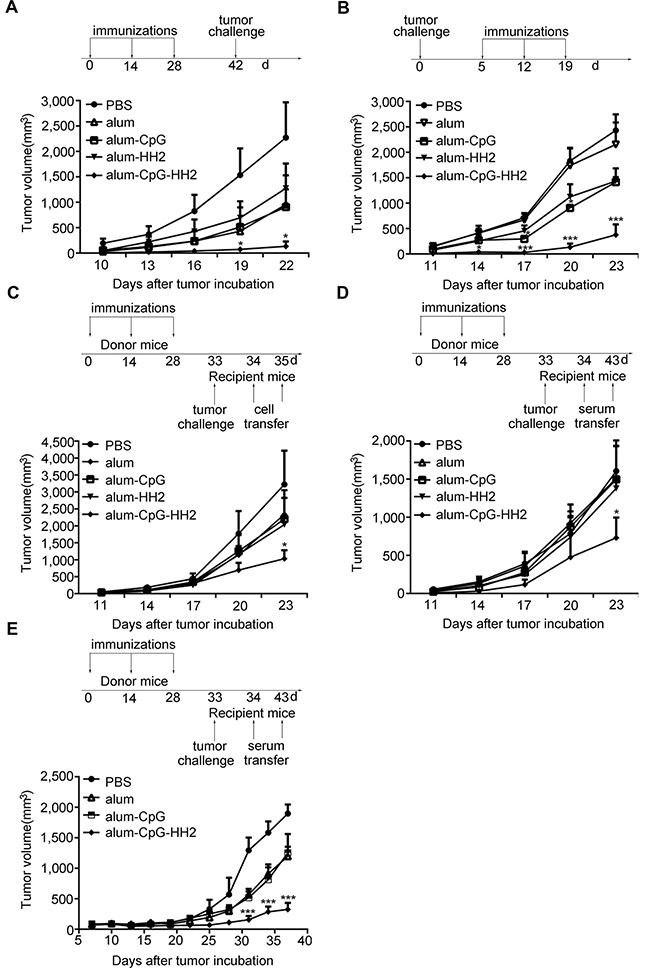
Enhanced anti-tumor immunity by alum-CpG-HH2-NY vaccine in melanoma models (**A**) A comparison of the tumor growth in B16-bearing mice was performed in the prophylactic model (10 mice/group). Error bars represent mean + SEM. *p < 0.05. (**B**) In a therapeutic model, mice were inoculated with 2 × 10^5^ NY-ESO-1^+^ B16 cells and treated with indicated vaccines on days 5, 12 and 19 (6 mice/group). Error bars represent mean + SEM. ***p < 0.001. (**C** and **D**) The adaptive transfer experiments were conducted. Donor animals were treated with indicated vaccines on days 0, 14, and 28. Then the serum or splenocytes were prepared one week after the third immunization. Recipient mice (6 per group) were challenged with 2 × 10^5^ NY-ESO-1^+^ B16 cells on day 33 and i.v injected with 2 × 10^7^ splenocytes from donor animals on days 34 and 35 (**C**) or 0.1 ml of the sera from donor animals on days 34 to 43 (**D**). Error bars represent mean + SEM. *p < 0.05. (**E**) Tumor volume curves in female nude mice-bearing human A375 cells after injection with the sera from donor animals. Error bars represent mean + SEM. ***p < 0.001.

Next, we tested the anti-tumor effect of the alum-CpG-HH2 combinational adjuvant in mouse melanoma models. In the prophylactic model (Figure 2A), B16-bearing mice treated with PBS (control) displayed rapid tumor growth, with a high tumor volume of 2,270 mm^3^ on day 22 after tumor induction. Vaccination with alum-NY, alum-CpG-NY or alum-HH2-NY had some inhibitory effects on B16-melanoma growth, but showed no significant difference with PBS treatment. Interestingly, vaccination with alum-CpG-HH2-NY significantly blocked the tumor growth, having an average tumor volume of 132 mm^3^ on day 22 (*p*< 0.05 versus PBS). Moreover, no significant body weight changes or obvious pathological changes of the heart, liver, spleen, lung and kidney were observed in mice treated with any of the vaccine formulations (Supplementary Figure 1).

In the therapeutic model (Figure 2B), mice bearing NY-ESO-1^+^ B16 cells were employed. The tumor growth was progressive in a single treatment with PBS or alum-NY (mean volume, 2,431 mm^3^ and 2,153 mm^3^, respectively) and slightly attenuated in the treatment with alum-CpG-NY or alum-HH2-NY (mean volume, 1,413 mm^3^ and 1,434 mm^3^, respectively). In contrast, vaccination with alum-CpG-HH2-NY drastically blocked the tumor growth (mean volume, 375 mm^3^). Similar results were observed in the Hepa 1-6 hepatoma model (Supplementary Figure 2).

To investigate whether alum-CpG-HH2-NY could stimulate anti-tumor immunity in an adoptive cellular or serum therapy model, we transferred the splenocytes or serum from the different vaccinated C57BL/6 mice to recipient C57BL/6 mice bearing NY-ESO-1^+^ B16 cells, using the protocols shown in Figures 2C and 2D. Tumors grew progressively in PBS group, and there was moderate inhibitory effect on tumor growth in alum-NY, alum-HH2-NY or alum-CpG-NY groups. In contrast, tumor growth in alum-CpG-HH2-NY group was markedly inhibited.

To further evaluate the effect of passive serum therapy on human A375-mln1.luc tumor model, A375-bearing nude mice were administered with serum from vaccines-treated or naïve C57BL/6. A significant delay in tumor growth was observed in the alum-CpG-HH2-NY-treated mice compared to other groups (Figure 2E). Taken together, these results demonstrate that NY-ESO-1 vaccine with alum-CpG-HH2 combinatorial adjuvant has much more potent anti-tumor activity *in vivo* than that with alum, alum-CpG or alum-HH2.

### Antigen-specific humoral immune response is strongly stimulated by alum-CpG-HH2-NY vaccine

**Figure 3:**
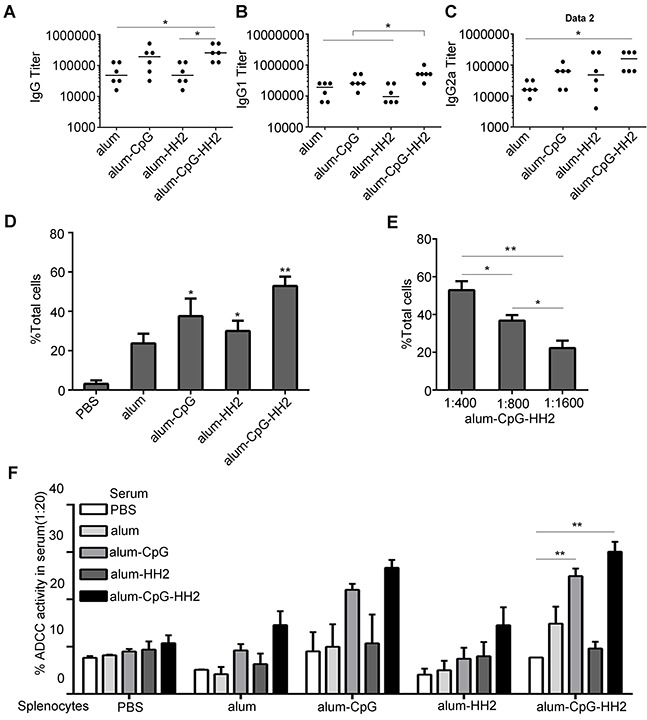
NY-ESO-1-specific antibody response induced by alum-CpG-HH2-NY vaccine (**A-C**) ELISA was used to detect the titers of total IgG, IgG1 and IgG2a of anti-NY-ESO-1 antibodies in the treated groups, respectively. Error bars represent median. (**D**) The NY-ESO-1-specific IgG antibodies binding NY-ESO-1^+^ B16 melanoma cells were determined by FACS analysis (n = 3 mice). Error bars represent mean + SEM. *p < 0.05, **p < 0.01. (**E**) Specificity of anti-NY-ESO-1 antibodies from the serum against NY-ESO-1 was detected by FACS analysis (n= 3 mice). Error bars represent mean + SEM. *p < 0.05, **p < 0.01. (**F**) Antibody-dependent cellular cytotoxicity was determined using the ^51^Cr-release assay (n= 3 mice). Error bars represent mean + SEM. **p < 0.01.

The antigen-specific antibody triggered by the various vaccines was assessed one week after the third immunization in prophylaxis against B16 melanoma. We found that alum-CpG-NY vaccine elevated the titers of IgG and IgG2a slightly but not significantly, compared to alum-NY or alum-HH2-NY vaccines. On the contrary, mice receiving alum-CpG-HH2-NY vaccine had significantly higher IgG and IgG2a titers than those receiving alum-NY vaccine (*p* < 0. 05) (Figures 3A-3C). Moreover, alum-CpG-HH2-NY vaccine induced a considerable higher IgG1 titers than other three groups (*p* < 0. 05) (Figure 3B). In addition, antibody binding to NY-ESO-1^+^B16 melanoma cells was detected by flow cytometry. As expected, the largest amount of antibody was observed in the alum-CpG-HH2-NY group at serum dilution ratio 1:400 (Figure 3D). Moreover, alum-CpG-HH2-NY group displayed an increasing serum binding positive index of NY-ESO-1^+^B16 with a reduced serum dilution ratio (Figure 3E), suggesting that the combination between NY-ESO-1^+^B16 cells and antibody had high specificity. To analyze the *ex vivo* ADCC activity of anti-NY-ESO-1 antibody, NY-ESO-1^+^ B16 melanoma cells were exposed to serum from different groups followed by the incubation with splenocytes from PBS or vaccines-treated groups. As shown in Figure 3F, the highest ADCC activity *ex vivo* occurred when both serum and splenocytes came from alum-CpG-HH2-NY group. The results suggest that alum-CpG-HH2-NY vaccine strongly stimulates antigen-specific humoral immune response.

### Alum-CpG-HH2-NY vaccine induces a potent NK cell cytotoxicity and CTLs activity

**Figure 4:**
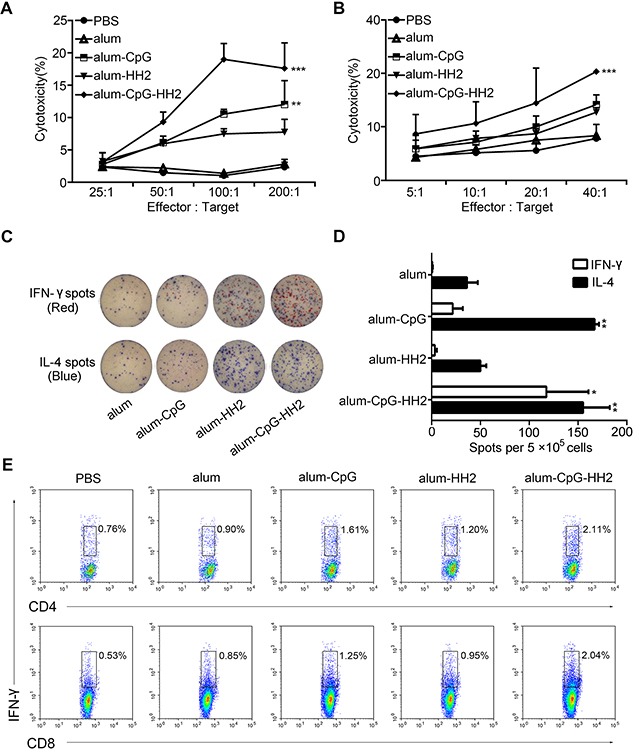
NK cytolytic activity and specific cellular response elicited by alum-CpG-HH2-NY vaccine (**A**) The 4-h NK assay against YAC-1 targets was determined at various effector:target ratios. Error bars represent mean + SEM. **p < 0.01, ***p < 0.001. (**B**) The cytolytic activity of splenocytes against NY-ESO-1^+^ B16 cells was measured using a 4 h ^51^Cr-release assay at various E:T ratios. Error bars represent mean + SEM. ***p < 0.001. (**C**) The representative graphs of spot-forming cells for IFN-γ and IL-4 at one week post-immunization in the ELISpot assay. (**D**) The average number of IL-4/IFN-γ secreting splenocytes was calculated (n = 3, three independent experiments). Error bars represent mean + SEM. *p < 0.05, **p < 0. 01. (**E**) T cell analysis in splenocytes after vaccination. Intracellular staining of IFN-γ in CD4^+^and CD8^+^T cells was analyzed by FACS.

Stimulated and activated NK cells occur predominantly during the early phases of the immune response, playing a crucial role in anti-tumor immunity. Next, the NK cell activity from each group at 48 h after the first immunization was compared at different effector : target ratio (25:1, 50:1, 100:1, and 200:1). As shown in Figure 4A, no significant NK cell activity against YAC-1 cells was found in animals vaccinated with PBS, alum-NY, or alum-HH2-NY at different E:T ratios, while high NK cytotoxicity was detected from alum-CpG-NY group, compared with the above-mentioned groups (*p* < 0.05). The NK cytolytic activity was even higher in alum-CpG-HH2-NY group than in the alum-CpG group (*p* < 0.001), and a maximal cytotoxicity was acquired using 100:1 ratio. The results support that alum-CpG-HH2-NY vaccine induces a potent NK cell cytotoxicity.

To assess the CTLs activity induced by alum-CpG-HH2-NY vaccine, splenocytes were activated and employed as effectors to recognize NY-ESO-1^+^B16 melanoma cells. PBS, alum-NY, alum-CpG-NY and alum-HH2-NY induced no or moderate CTLs activity. In contrast, alum-CpG-HH2-NY vaccine induced remarkable tumor antigen-specific cytotoxic activity (*p* < 0.001) (Figure 4B). In addition, the number of spot-forming cells (SFCs) for IFN-γ and IL-4 was evaluated by ELISpot. As shown in Figures 4C and 4D, the number of IL-4 SFCs in alum-CpG-NY group was significantly higher than that in alum-NY or alum-HH2-NY group. Addition of HH2 to alum-CpG-NY vaccine did not further increase the number of IL-4 SFCs, compared to alum-CpG-NY. Interestingly, there was a significant elevation of IFN-γ SFCs in alum-CpG-HH2-NY group compared with alum-CpG-NY group (*p* < 0.05). These observations were further confirmed by analyzing the levels of IFN-γ-secreting CD4^+^/CD8^+^T cells in each group. Similarly, the proportion of NY-ESO-1 specific IFN-γ-secreting CD4^+^/CD8^+^T cells was significantly higher in alum-CpG-HH2-NY group than in other groups (Figure 4E). Collectively, the results indicate that alum-CpG-HH2-NY vaccine induces a potent CTLs activity.

### Alum-CpG-HH2-NY vaccine increases lymphocytes infiltration into tumors

**Figure 5:**
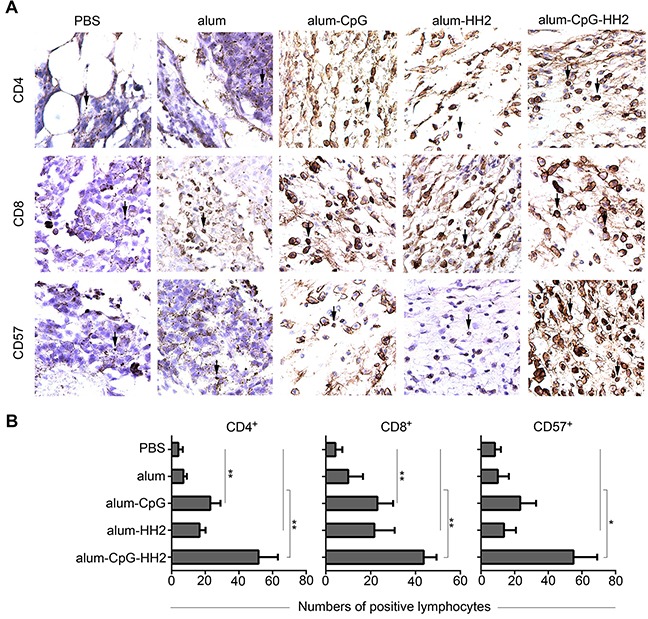
Lymphocyte infiltration in mice melanoma model by immunohistochemistry analyses (**A**) A part of the whole graph from IHC staining for lymphocyte infiltration in each group (box-marked graphs from Supplementary Figure 3). Serial sections of tumor tissue were stained with anti-CD4, anti-CD8 and anti-CD57 mAbs at 4°C overnight. Afterwards, sections were exposed to anti-mouse biotinylated antibody and streptavidin-biotinylated orseradish peroxidase complex. (**B**) The semi-quantification of CD4^+^, CD8^+^and CD57^+^ lymphocytes in the tumor tissues was performed. The arrows indicate the positive lymphocytes. Error bars represent mean + SEM. *p < 0.05, **p < 0.01.

Infiltration of CD4^+^, CD8^+^, and CD57^+^ lymphocytes in tumors was investigated by IHC analyses. As shown in Figures 5A and 5B, the proportions of CD4^+^, CD8^+^, and CD57^+^ lymphocytes were small in both PBS and alum-NY groups but slightly increased in alum-HH2-NY group. CD4^+^, CD8^+^, and CD57^+^ lymphocytes were abundant in alum-CpG-NY group. By statistical analysis, we found that the proportions of CD4^+^ and CD8^+^ lymphocytes were significantly increased in alum-CpG-NY group compared to PBS group. Importantly, the proportions of CD4^+^, CD8^+^, and CD57^+^ lymphocytes were very significantly higher in tumors from the alum-CpG-HH2-NY group compared to almost all other groups (Figure 5).

### The protection of alum-CpG-HH2-NY-immunized mice is impaired in the absence of NK cells and CD8^+^ T cells

**Figure 6:**
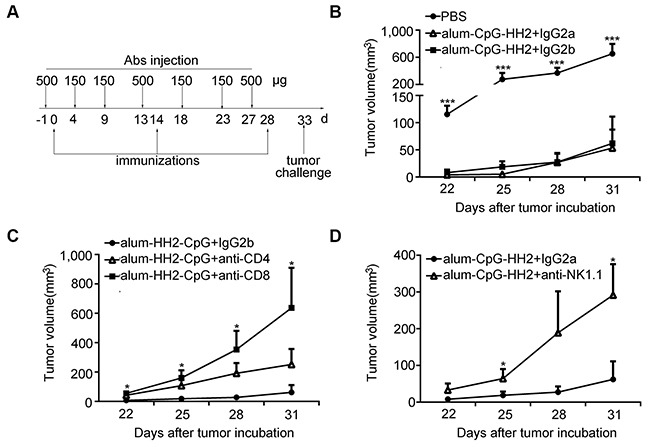
Impaired protection of alum-CpG-HH2-NY-immunized mice by depletion of the immune cell subsets (**A**) A representative schedule of immune cell subsets depletion (described in *Materials and Methods*). (**B**-**D**) The curve graphs exhibited the tumor growth of each group (6 per group) and the abrogation of the anti-tumor immunity was examined in the absence of CD4^+^ T cells, NK cells and CD8^+^ T cells. Error bars represent mean + SEM. *p < 0.05, ***p < 0.001.

Our above findings that alum-CpG-HH2-NY vaccine was able to enhance humoral immunity, induce NK cell activity and activate CTLs activity suggest that CD4^+^ T, NK and CD8^+^ T cells may play an important role in anti-tumor immunity. Next, we examined the anti-tumor response induced by alum-CpG-HH2-NY vaccine in the absence of CD4^+^ and CD8^+^ T and NK cells, using the protocol shown in Figure 6A. Treatment with rat IgG2a or IgG2b had no or little impairment of protection elicited by alum-CpG-HH2-NY vaccine (Figure 6B). Depletion of CD8^+^ T cells entirely abrogated the anti-tumor immunity of alum-CpG-HH2-NY, whereas depletion of CD4^+^ T cells slightly diminished the anti-tumor immunity and showed no statistical difference relative to IgG2b treatment (control) (Figure 6C). Depletion of NK cells significantly attenuated the anti-tumor immunity of alum-CpG-HH2-NY (Figure 6D). These results suggest that NK cells and CD8^+^ T cells play a critical role in anti-tumor activity of alum-CpG-HH2-NY vaccine, while CD4^+^ T cells may be a potent inducer of humoral immunity and make partial contribution to anti-tumor immunity of alum-CpG-HH2-NY.

## DISCUSSION

Despite extensive research and accumulating advances in cancer treatment in recent years, cancer is still the leading cause of death in most countries, indicating that it is urgently needed to develop novel and effective treatments. Cancer immunotherapy is regarded as a promising strategy to combat cancer [27]. On the way to cancer immunotherapy, cancer vaccines containing tumor-associated antigens and adjuvants have made exciting achievements in clinical settings [4]. NY-ESO-1, a cancer-testis antigen, is expressed in diverse human tumor types, but is not found in normal tissues except for germ cells and trophoblasts [28]. NY-ESO-1 is a prospective tumor antigen for clinical trials because of the high immunogenicity and tissue distribution [29, 30]. In this study, we demonstrated that alum-CpG-HH2 combinatorial adjuvant was able to enhance the anti-tumor activity of NY-ESO-1 vaccine in multiple tumor models.

Results from antigen uptake analysis showed that the CpG-HH2 combinatorial adjuvant effectively promoted the uptake of NY-ESO-1 antigen by dendritic cells *in vitro*. All adjuvants make certain contributions to antigen presentation including antigen uptake by antigen-presenting cells (APC), activation of APC and expression of co-stimulatory molecules in a direct or indirect manner [31]. The functions of dendritic cells described above play a central regulatory role in the initiation of tumor immunity [32]. Moreover, the maturation of DCs by up-regulating the expression of surface markers CD80, CD86 and MHCII has been reported in our previous researches [26].

It has been described that CpG specifically induces the activation of TLR9, resulting in various biological functions, in which the activation of MAPK and NF-κB was required [33, 34]. Cationic antimicrobial peptides, such as LL-37, can also activate Erk1/2, p38 or NF-κB [35]. In this study, the stronger promotion in the activation of NF-κB and the phosphorylation of Erk1/2 and p38 was observed in the BMDCs stimulated with CpG-HH2 combination than single component. Molecular pathways of alum in dendritic cells and macrophages have been described [36] as follows. The binding of alum to cell membrane induces the formulation of lipid raft which activates the ITAM-Syk-PI3Kδ pathway followed by the activation of p38 MAP kinase. Furthermore, the release of dsDNA induced by alum can activate NF-κB. Therefore, we speculate that alum-CpG-HH2 induced strong activation of p38 MAP kinase and NF-κB, which might be associated with its enhanced anti-tumor immunity. Undoubtedly, the precise molecular mechanism behind the anti-tumor immunity of the combinational adjuvant remains to be further studied.

Natural killer (NK) cells are the primary effector lymphocytes of the innate immune response [37]. Activated NK cells can also induce the production of IFN-γ, TNF-α, and a variety of important cytokines and chemokines [38]. CpG is a potent stimulator of NK cells [39]. NK cell cytotoxicity occurs when multiple adjuvants are present. As expected, alum-CpG-NY was effective in activating NK cells compared to PBS, alum-NY or alum-HH2-NY. However, interestingly, alum-CpG-HH2-NY vaccine was able to induce the highest NK cell killing capacity, among all adjuvants tested in this study. Depletion of NK cells partially attenuated the anti-tumor immunity induced by alum-CpG-HH2-NY vaccine, suggesting that NK cells may be involved in the early phases of generating anti-tumor immunity [40].

In recent years, vaccine adjuvant design inducing Th1 type immune response has been a promising strategy for cancer vaccines [3, 41–43]. The production of IFN-γ and TNF-α was examined in this study. Our results revealed that CpG combined with HH2 markedly enhanced the expression of IFN-γ in PBMCs *in vitro* and showed a synergistic effect compared to CpG or HH2 alone. The up-regulation of TNF-α by CpG- HH2 combination was statistically significant but not synergistic. This fact strongly suggests that Th1 type immune response was driven by the enhanced secretion of Th1-type cytokines, which typically activate CTLs, monocytes, NK cells, macrophages to fight tumor [44, 45]. CTLs are critical effector cells in anti-tumor immune responses by specifically recognizing their target cells and secreting cytolytic contents [46]. The strongest stimulation of the CTLs activity of spleen cells was observed in mice treated with alum-CpG-HH2-NY vaccine. Moreover, compared to other four groups, alum-CpG-HH2-NY vaccine induced a highest level of IFN-γ-secreting CD8^+^ T cells. Importantly, depletion of CD8^+^ T cells completely abrogated the anti-tumor immunity of alum-CpG-HH2-NY vaccine, indicating that CD8^+^ T cells is a very crucial fighter against tumor cells.

In this study, we also examined humoral immune response and lymphocyte infiltration in mice treated with various adjuvant combinations *in vitro*. Mice treated with alum-CpG-HH2-NY vaccine had the highest titers of IgG, which may be related to the significant induction of ADCC against NY-ESO-1^+^ B16 cells. ADCC activity, one of the critical parameters for vaccine design or efficacy of therapeutic antibodies, is capable of directly triggering apoptosis of target cells [47, 48]. Depletion of CD4^+^ T cells weakly attenuated the anti-tumor effect of alum-CpG-HH2-NY vaccine, supporting that CD4^+^T cells might be a potent inducer of humoral immunity and required for CD8^+^ CTL response[49]. In addition, the preponderance of CD4, CD8, and CD57-positive lymphocytes being accessing to the tumor cells in the group immunized with alum-CpG-HH2-NY vaccine may also make certain contributions to tumor regression.

These above actions involve enhanced cytokines production, activated NK cell activity and CTL activity, as well as elevated humoral immunity and lymphocyte infiltration, all of which may contribute to the anti-tumor activity of alum-CpG-HH2-NY vaccine. In conclusion, our data demonstrated that alum-CpG-HH2 combination is a safe, effective and promising adjuvant combination, which can effectively trigger innate immunity, modulate humoral immune response and especially stimulate Th1 type immune response to suppress the tumor growth. The alum-CpG-HH2 combinatorial adjuvant lays a foundation for a novel approach for cancer therapy.

## MATERIALS AND METHODS

### Mice and cell lines

Female C57BL/6 (6-8 weeks old) and nude mice (4-6 weeks old) were provided by Huafukang Biological Technology Company (Beijing, China). All mice were maintained under SPF conditions and in accordance with protocols approved by the Ethics Review Committee for Animal Experimentation of Sichuan University. NY-ESO-1^+^ B16-F10 melanoma cells and NY-ESO-1^+^ Hepa1-6 cells stably expressing human NY-ESO-1 were prepared in our lab as described previously [16], and cultured in Dulbecco's modified Eagle's medium (DMEM, Gibco, Grand Island, USA) supplemented with 10% fetal bovine serum (FBS) (Gibco, Grand Island, USA) and 800 μg/ml of G418 (Sigma-Aldrich, St. Louis, MO, USA).

### Mouse models

Preparations of NY-ESO-1 protein and vaccines are shown in Supplementary Material. In the prophylactic model, C57BL/6 mice were subcutaneously (s.c.) vaccinated with alum-NY, alum-CpG-NY, alum-HH2-NY, or alum-CpG-HH2-NY on days 0, 14, and 28. PBS was used as a control. NY-ESO-1^+^ B16 (2×10^5^) cells were implanted on the back of mice two weeks after the last immunization and tumor volume was measured every three days from tumor onset (∼3 mm diameter). Tumor volume was calculated according to the formula: tumor volume = 0.5 ×length (mm) × [width (mm)]^2^.

For the therapeutic tumor experiment, C57BL/6 mice were challenged with a s.c. injection of 2×10^5^ NY-ESO-1^+^ B16 tumor cells. On day 5 after tumor cell injection, the tumor was palpable (>50 mm^3^). Mice were then randomized into 5 groups and vaccinated once a week for three times (on days 5, 12, and 19).

In the adoptive transfer settings, C57BL/6 mice were treated with the above vaccines for a total of three injections and splenocytes or serum were collected one week after the third immunization. B16-bearing mice were challenged with an intravenous injection of 2×10^7^donor splenocytes twice (once per day) or 0.1 ml serum for 10 consecutive days on the second day of tumor inoculation. Alternatively, nude mice were challenged with 5×10^6^ human melanoma A375 cells (ATCC, Rockville, USA) and then subjected to passive serum therapy as described above.

### Analysis of NY-ESO-1 antibody titers

Antigen-specific antibodies in the serum of one week after the third immunization were determined using ELISA. In short, 2-fold diluted serum was analyzed on 96-well plates (Nunclon, Roskilde, Denmark) coated with 0.1 μg NY-ESO-1 protein per well. NY-ESO-1-specific antibodies were probed with goat anti-mouse IgG, IgG2a or IgG1 conjugated with horseradish peroxidase (1:3000; ZSGB-BIO, Beijing, China). Finally, plates were read on an ELISA reader at a wavelength of 450 nm.

To determine the specificity of antibodies, serial dilutions of serum (1:400, 1:800, and 1:1600) were incubated with 3×10^5^ NY-ESO-1^+^ B16 cells for 1 h at 4°C after fixation and permeabilization. The cells were then washed in PBS, stained with FITC-conjugated goat anti-mouse IgG (BD Pharmingen, San Jose, CA) for 30 min at 4°C and analyzed using BD FACS Calibur flow cytometry (BD Pharmingen).

### Detection of ADCC, NK and CTLs activities

In these experiments, YAC-1 or NY-ESO-1^+^B16 cells were labeled with 100 μCi ^51^Cr and then used as target cells. In ADCC assay, 1×10^6^ NY-ESO-1^+^B16 cells were exposed to a 1:20 diluted serum followed by labelling with ^51^Cr at 37°C for 1 h. After washing, 1×10^4^ ^51^Cr-labelled NY-ESO-1^+^B16 cells were co-incubated with 1×10^6^ splenocytes of one week after the third immunization at 37°C for 4 h. To detect NK activity, splenocytes were collected at 48 h after the first vaccination and incubated with ^51^Cr-labelled YAC-1 cells at serial effector:target ratios (25:1, 50:1, 100:1, and 200:1) at 37°C for 4 h. For CTLs assay, splenocytes were isolated one week after the third immunization, sensitized with 10 μg/ml NY-ESO-1 protein at 37°C for 16 h, and incubated with ^51^Cr-labelled NY-ESO-1^+^B16 cells at different effector:target ratios (5:1, 10:1, 20:1, and 40:1). Finally, the supernatants were collected and the released radioactivity was measured using a gamma counter (LKB Wallac, Turku, Finland). Percent lysis was determined according to this formula: (test lysis – spontaneous lysis) / (maximum lysis – spontaneous release) ×100.

### ELISpot assay and IFN-γ intracellular staining

Splenocytes isolated from PBS- or vaccines-immunized mice one week after the last immunization were subjected to ELISpot assay and IFN-γ intracellular staining. The mouse IFN-γ/IL-4 Dual-Color ELISpot kit (R&D Systems, Minneapolis, USA) was used for ELISpot assay. Briefly, 5×10^5^ splenocytes were co-incubated with 10 μg/ml NY-ESO-1 in the mouse IFN-γ-specific monoclonal antibody and IL-4-specific polyclonal antibody pre-coated microplates at 37°C for 48 h. Next, an enzyme-linked colorimetric assay was carried out for further detection. The viable cells were quantified using an ELISpot reader system (Beijing Dakewe Biotech Company, Beijing, China).

For IFN-γ intracellular staining, splenocytes were stimulated in the presence of 10 μg/ml NY-ESO-1 at 37°C for 1 h and then incubated in Golgi Plug for an additional 6 h. After staining with PerCP-anti-mouse CD4 and PE-anti-mouse CD8α (BD Pharmingen), splenocytes were fixed and permeabilized using a Cytofix/Cytoperm Kit (BD Pharmingen) according to manufacturer's instructions. The intracellular IFN-γ was detected with FITC-anti-mouse IFN-γ (BD Pharmingen) and analyzed on a BD FACS Calibur flow cytometer.

### Immunohistochemistry(IHC)

IHC was conducted using a standard method as previously described [16].

### Cytokines assay

Human peripheral blood mononuclear cells (PBMCs) were isolated from healthy volunteers. PBMCs (5×10^5^ per well) were stimulated with 20 μg CpG, 40 μg HH2 or 20 μg CpG in combination with 40 μg HH2 for 24 h. The supernatants were collected for detecting the levels of IFN-γ, TNF-α and IL-1β using Quantikine® Human IFN-γ, TNF-α and IL-1β ELISA kits (R&D Systems).

### Antigen uptake

Bone marrow cells were isolated from C57BL/6 mice and cultured in the presence of 10 ng/ml recombinant murine GM-CSF and IL-4 (10ng/ml; both from PeproTech, Rocky Hill, NJ). Bone marrow-derived dendritic cells (BMDCs) from 5-day-old cultures were gathered for further experiments. CpG (20 μg/ml), HH2 (40 μg/ml), or CpG-HH2 combination were mixed with Alexa Fluor 488-labeled NY-ESO-1 (10 μg/ml) for complex formation at 37°C for 15 min. Then BMDCs (2 ×10^5^ per well) were pulsed with Alexa Fluor 488-labelled NY-ESO-1 or NY-ESO-1/complex adjuvant formulations for 3 h at 37°C. At the end of the incubation, cells were washed, fixed, stained with DAPI (Beyotime, Haimen, China) and examined under a confocal microscope.

### Western blotting

BMDCs were incubated with CpG (20 μg/ml), HH2 (40 μg/ml), or CpG-HH2 complex for 30 and 45 min, respectively. Protein extracts were prepared from treated-DCs, separated by 12% SDS-PAGE and transferred onto polyvinylidene fluoride membranes (Millipore, Billerica, MA). The membranes were blocked with 5% non-fat milk and probed with rabbit antibodies against p65, phospho-p65, p38, phospho-p38, Erk1/2 and phospho-Erk1/2 (1:2000; all from Cell Signaling Technology, Beverly, MA) overnight at 4°C. Then the membranes were incubated with horseradish peroxidase-conjugated goat anti-rabbit IgG (1:5000; ZSGB-BIO, Beijing, China).

### *In vivo* immune cell subsets depletion

The depletion of CD4^+^ T cells, CD8^+^T cells and NK cells *in vivo* was conducted as described [49]. Animals were treated with alum-CpG-HH2-NY vaccine on days 0, 14, and 28, and challenged with NY-ESO-1^+^ B16 cells (2×10^5^ cells) one week after the third immunization. Mice were injected i.p. with 500 μg of either the antibodies [anti-CD4 (clone GK 1.5, rat IgG2b), anti-CD8 (clone 2.43, rat IgG2b), anti-NK1.1 (clone PK136, or rat IgG2a)], or isotype controls (rat IgG2a or IgG2b) one day prior to each vaccine immunization, and then with 150 μg of the antibodies twice (5 days once) until next vaccine immunization. PBS served as a control. The depletion of CD4^+^ T cells, CD8^+^T cells and NK cells was determined by flow cytometry.

### Statistical analysis

All statistical analyses were performed using SPSS 16.0 software (SPSS Benelux, Gorinchem, The Netherlands). Comparisons for multiple groups were performed using one-way analysis of variance (ANOVA) test followed by Tukey's multiple-range testing. Values of *p* < 0.05 were considered statistically significant.

## SUPPLEMENTARY MATERIALS FIGURES AND TABLES



## Abbreviations

ADCC: antibody-dependent cellular cytotoxicity
Alum: aluminum salts
APC: antigen-presenting cells
BMDCs: bone marrow-derived dendritic cells
CpG: CpG oligodeoxynucleotide
CTLs: cytotoxic T lymphocytes
IDRs: innate defense regulators
IHC: immunohistochemistry
PBMCs: human peripheral blood mononuclear cells
PBS: phosphate-buffered saline
SFCs: spot-forming cells
TLR9: Toll-like receptor 9.
