# Supplementary material for: The novel complex combination of alum, CpG ODN and HH2 as adjuvant in cancer vaccine effectively suppresses tumor growth in vivo
Source: Oncotarget. 2017 Apr 28;8(28):45951–64. doi: 10.18632/oncotarget.17504 (PMC5542240; doi:10.18632/oncotarget.17504)
Supplement: Supplementary file 1 [file oncotarget-08-45951-s001.pdf]

The novel complex combination of alum, CpG ODN and HH2 as adjuvant in cancer vaccine effectively suppresses tumor growth *in vivo*

Supplementary Materials

MATERIALS AND METHODS

Preparation of NY-ESO-1 protein and vaccines

NY-ESO-1 protein (18 kDa) was prepared according to the protocols from a previous study [16]. Finally, the quality of NY-ESO-1 protein was detected by SDS-PAGE and Western blotting (Supplementary Figure 4).

To prepare the vaccines, 20 µg CpG 1826 (Thermo Fisher Scientific, San Jose, CA, USA) was incubated with 40 µg HH2 (Ketai Biotechnology Company, shanghai, China) to form stable CpG-HH2 complex and then mixed with 125 µg alum (Brenntag Biosector, Frederikssund, Denmark) at 37°C for 10 min, followed by the addition of 5 µg recombinant NY-ESO-1 protein. All vaccine formulations were in a total volume of 100 µL with phosphate-buffered saline (PBS, pH = 7.4). Mice in each tumor model reported here were divided into five groups. The clear representation of vaccine formulations in each group was shown in Supplementary Table 1.

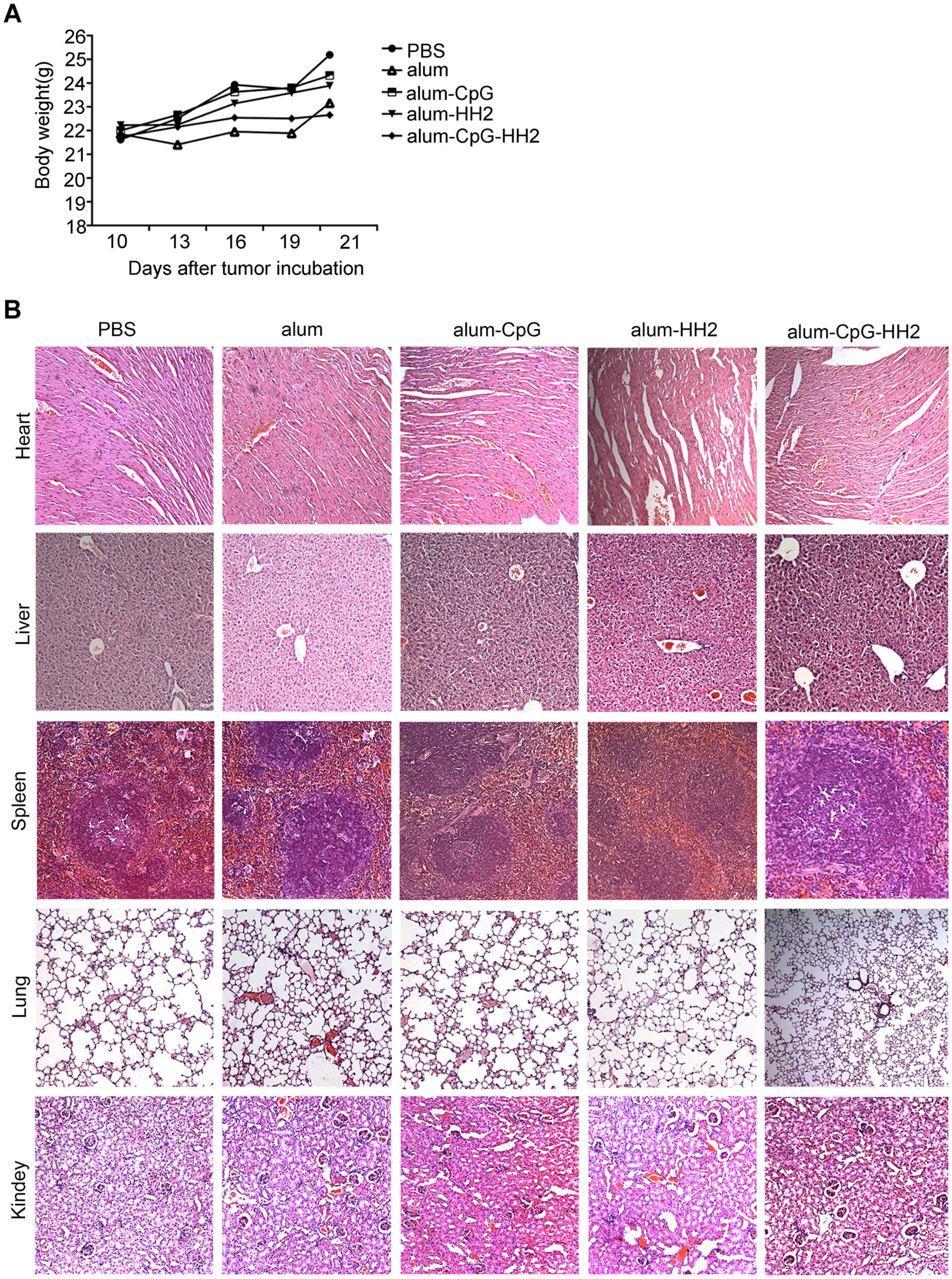

Supplementary Figure 1: Body weight and histological analyses of all major organs. (**A**) Body weight was determined every 3 days during the delivery period. (**B**) Histological analyses of heart, liver, spleen and kidney were performed by H&E staining (200×magnification).

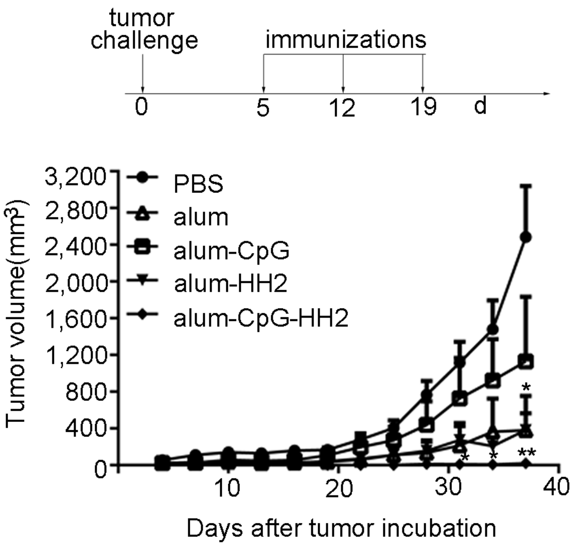

Supplementary Figure 2: Alum-CpG-HH2-NY vaccine inhibits tumor growth in therapeutic hepatoma models. Mice were inoculated with 1 × 10^7 NY-ESO-1^+ Hepa1-6 cells and treated with indicated vaccines on days 5, 12 and 19. Error bars represent mean + SEM. **p* < 0.05, ***p* < 0.01.

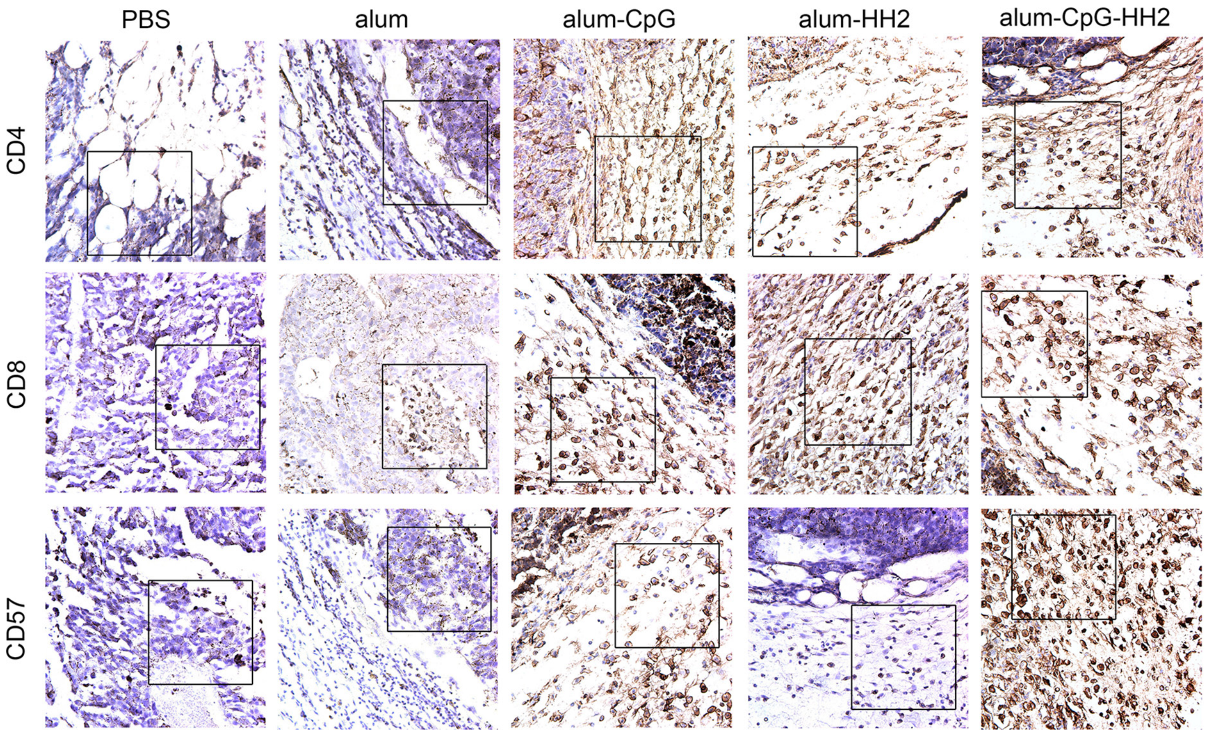

Supplementary Figure 3: The representative IHC staining for $CD4^+$, $CD8^+$, and $CD57^+$ lymphocytes in tumor tissue from each group. A square portion of the whole image was selected and enlarged for a better observing of the positive lymphocytes.

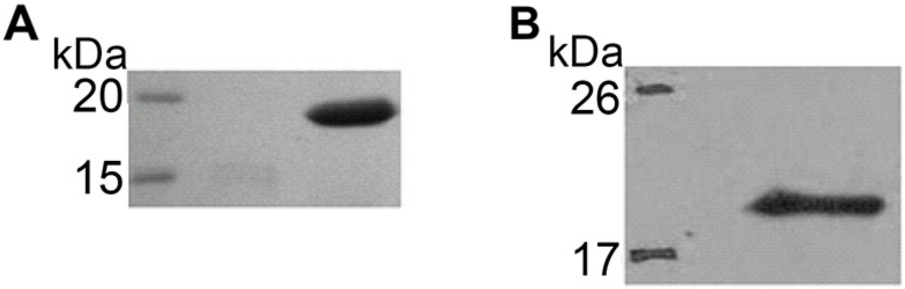

Supplementary Figure 4: The identification of NY-ESO-1 protein. (**A**) SDS-PAGE analysis of purified NY-ESO-1 protein. (**B**) Western blot analysis of purified NY-ESO-1 protein, showing that NY-ESO-1 specifically binds to anti-human NY-ESO-1 antibody.

Supplementary Table 1: The formulations of vaccines used in animal experiments

Vaccines or groups	Vaccine formulations			
	**Recombinant NY-ESO-1 protein (NY)**	**CpG**	**HH2**	**alum**
alum-NY	5 μg	/	/	125 μg
alum-CpG-NY	5 μg	20 μg	/	125 μg
alum-HH2-NY	5 μg	/	40 μg	125 μg
alum-CpG-HH2-NY	5 μg	20 μg	40 μg	125 μg

Note: All vaccines were prepared to a total volume of 100 μL with PBS (pH = 7.4).
